# Sal is a proteobacterial bile acid aldolase that repurposes key thiolase catalytic residues for retroaldol cleavage of C_5_ steroid side chains

**DOI:** 10.1016/j.jbc.2025.110439

**Published:** 2025-07-01

**Authors:** Nicolas Rolfe, Kurt L. Schroeter, Taylor JB. Forrester, Matthew S. Kimber, Stephen YK. Seah

**Affiliations:** Department of Molecular and Cellular Biology, University of Guelph, Guelph, Ontario, Canada

**Keywords:** aldolase, bile acids, enzyme complex, steroids, thiolase

## Abstract

Aldolases hold potential as biocatalysts for the synthesis of novel steroid pharmaceuticals. The steroid aldolase from *Comamonas testosteroni* (*Ct*Sal) forms a complex with *C. testosteroni* steroid hydratase (*Ct*Shy). *Ct*Sal cleaves the C_5_ side chain of bile acid thioester steroids, whereas a previously characterized actinobacterial homolog from *Thermonospora curvata* (*Tc*Ltp2) targets the C_3_ side chain. We identified Tyr302 and Cys304 as the catalytic residues in *Ct*Sal, different from the paired Tyr residues found in *Tc*Ltp2. The 1.95 Å structure of *Ct*Sal bound to the C-terminal domain of unknown function 35 (DUF35) of *Ct*Shy (*Ct*Shy_**DUF35**_–*Ct*Sal) reveals a central *Ct*Sal dimer flanked by two *Ct*Shy_**DUF35**_ domains in an **αββα** arrangement. *Ct*Shy_**DUF35**_ has a unique Cys_3_His_1_ (C_3_H_1_) zinc finger that shapes the substrate-binding cleft of *Ct*Sal, preventing the binding of the flat cholesterol rings while accommodating the bent rings of bile acids. Phylogenetically, Sals and Ltp2s form separate clades and are distantly related to thiolases. Intriguingly, a *Trypanosoma brucei* homolog, annotated as a thiolase-like protein (*Tb*SLP), shares the catalytic architecture of *Ct*Sal, suggesting an aldolase rather than a thiolase function. This study provides the first detailed characterization of a C_5_ side chain steroid aldolase, revealing its unique catalytic features and expanding our understanding of steroid side chain catabolism in Proteobacteria.

Microbial steroid transformation has gained significant attention in recent decades due to its dual relevance to human health and environmental sustainability. In the gut microbiome, bile acid transformations generate steroid metabolites that regulate critical physiological processes that influence systemic host physiology (1). Conversely, environmental steroid endocrine disruptors such as estrone and estriol in municipal wastewater signify the urgent need for microbial degradation strategies to reduce ecological harm (2). Steroids are also an important class of therapeutics, ranking as the second-largest class of pharmaceuticals by market value, following antibiotics (3). Microbial transformation of steroids has become a cornerstone of steroid pharmaceutical production, as *de novo* chemical synthesis is often multi-step, expensive, and reliant on hazardous reagents, with additional challenges in achieving regio- and stereoselectivity. In contrast, steroid-degrading enzymes from microbial sources are efficient, regiospecific, and stereospecific, offering a promising alternative to traditional chemical synthesis methods, which often rely on chiral auxiliaries to achieve stereochemical control (3, 4, 5).

Steroids are recalcitrant to microbial degradation due to their intricate fused-ring structures, sparse functionalization, and poor aqueous solubility, which collectively impede enzymatic attack. Yet select Actinobacteria and Proteobacteria overcome these barriers, utilizing steroids as sole carbon sources *via* specialized catabolic pathways (6). Structurally, steroids are characterized by a tetracyclic sterane nucleus composed of three cyclohexane rings (A, B, and C) and one cyclopentane ring (D) (Fig. S1) (7). Substituent variations across the rings and side chains contribute to their structural diversity and facilitate their diverse biological roles in eukaryotes. For example, sterols like cholesterol, defined by a C3-OH group, regulate membrane fluidity and cell signaling, whereas bile acids such as cholate, with C3, C7, and C12 hydroxyl groups, are amphipathic, enabling the solubilization of dietary lipids (Fig. S1) (3, 8). While these structural features are critical for biological activity, they also complicate microbial degradation, particularly the cleavage of the extended side chain—an area requiring deeper exploration.

Bacterial degradation of steroid side chains shares mechanistic similarities with the β-oxidation of fatty acids. The process initiates with CoA ligase-catalyzed thioesterification of the carboxylate side chain, followed by sequential β-oxidation cycles, with the release of two or three carbon units per cycle as acetyl CoA or propionyl CoA (Fig. 1) (9). In Proteobacteria, C–C bond cleavage of bile acid side chains in each cycle occurs *via* a retroaldol reaction catalyzed by Sal and Ltp2 (Fig. 1) (10, 11, 12). Sal prefers C_5_ steroid side chains, while Ltp2 prefers C_3_ steroid side chains. Each aldolase forms a complex with the hydratase responsible for the preceding reaction in the pathway *via* their DUF35 domain (Fig. 1). Thus, Sal from *Comamonas testosteroni* KF1 interacts with the N-terminal DUF35 domain of the steroid hydratase *Ct*Shy, forming a hydratase–aldolase complex (hereby referred to as *Ct*Shy–*Ct*Sal) (13). Similarly, Ltp2 interacts with the heteromeric hydratase (ChsH1–ChsH2), forming the ChsH1–ChsH2–Ltp2 complex mediated by the DUF35 domain at the C-terminus of ChsH2 (ChsH2_DUF35_) (11, 12). The crystal structure of Ltp2 in complex with the DUF35 domain of ChsH2 from *Thermomonospora curvata* (hereby referred to as *Tc*ChsH2_DUF35_–*Tc*Ltp2) revealed a dimeric *Tc*Ltp2 adopting a thiolase fold, flanked by two DUF35 domains positioned on opposite sides of the dimer (12). In contrast, no crystal structure of Sal is currently available, and no Sal enzyme has been characterized beyond gene knockout studies.

**Figure 1:**
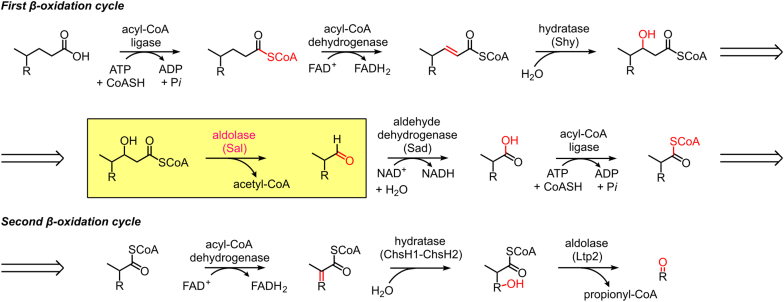
**Catabolism of the cholate side chain in Proteobacteria.** For simplicity, the steroid rings are denoted as R groups, with newly formed functional groups at each step highlighted in *red*. Reactions catalyzed by *Ct*Shy, *Ct*Sal, *Ct*Sad, *Ct*ChsH1–*Ct*ChsH2, and *Ct*Ltp2 are indicated in parentheses, with the reaction catalyzed by *Ct*Sal boxed in *yellow*.

Although *Tc*Ltp2 adopts a fold similar to sterol carrier protein-2 (SCP-2) type thiolases, its catalytic residues are distinct from those of canonical thiolases (12). A Tyr residue within the conserved YDHF motif acts as a catalytic base, abstracting a proton from the Cβ-hydroxyl group to initiate C–C bond cleavage. The His residue in this motif functions as an oxyanion hole, stabilizing the carbonyl oxygen of the thioester intermediate *via* hydrogen bonding (12). Following cleavage, a second Tyr residue in the catalytic site donates a proton to the enolate thioester intermediate. In Sal, however, this second catalytic Tyr is replaced by Gln, which is unlikely to act as a general acid, and the His in the YDHF motif is substituted with Cys.

Here, we report the biochemical and structural characterization of *Ct*Sal, addressing a gap in our understanding of steroid side chain catabolic pathways. While much is known about enzymes that degrade steroid side chains, Sal has not been well-characterized, and the molecular basis for its specificity for C_5_ steroid side chains remains unclear. By identifying critical residues for its activity through site-specific mutagenesis, our findings suggest that the catalytic residues of Sal and Ltp2 influence steroid side chain length specificity. Specifically, Sal appears to repurpose a Cys residue with minimal steric bulk from SCP-2 thiolase catalytic machinery for C_5_ side chain catalysis, while Ltp2 remodels the active site further with an extended Tyr residue to accommodate shorter C_3_ side chains. This work contributes to our understanding of bacterial steroid side chain C–C bond cleavage and could inform the design of more efficient biocatalysts for the synthesis of steroid pharmaceuticals.

## Results

### *Ct*Sal prefers bile acid substrates over cholesterol derivatives

**Figure 2:**
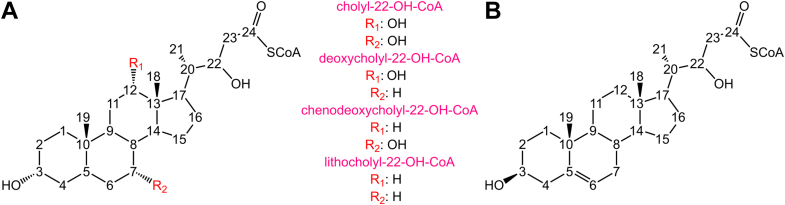
**Structure of steroid metabolites with C22 hydrated C_5_ side chains.***A*, bile acids cholyl-22-OH-CoA, deoxycholyl-22-OH-CoA, chenodeoxycholyl-22-OH-CoA, and lithocholyl-22-OH-CoA. *B*, cholesterol metabolite 3β,22-dihydroxy-chol-5-en-24-oyl-CoA.

Although recombinant *Ct*Sal can be purified in complex with just the DUF35 domain of *Ct*Shy with retention of aldolase activity, the complex rapidly loses activity upon dilution for kinetic assays (13). Consequently, *in vitro* analyses of *Ct*Sal were conducted using the full *Ct*Shy–*Ct*Sal complex. Using steady-state kinetics, the substrate specificity of *Ct*Sal within the full complex was assessed against a panel of modified bile acids and sterols, including cholyl- and cholesterol-derived CoA adducts (Table 1; Fig. S2). *Ct*Sal demonstrated broad specificity for bile acids with varying hydroxyl groups on the steroid ABC rings (Fig. 2*A*). Specifically, similar *k*_*cat*_*/K*_*m*_ values were observed for C_5_ side chain analogs: cholyl-22-OH-CoA (a primary bile acid derivative synthesized directly in the liver, bearing hydroxyl groups at C3, C7, and C12), deoxycholyl-22-OH-CoA (a secondary bile acid formed from cholic acid bearing C3 and C12 ring hydroxyl substituents), chenodeoxycholyl-22-OH-CoA (another primary bile acid bearing C3 and C7 ring hydroxyl substituents), and lithocholyl-22-OH-CoA (a secondary bile acid derived from bacterial processing of chenodeoxycholic acid bearing only a C3 hydroxyl). In contrast, the *k*_*cat*_*/K*_*m*_ value for 3β,22-dihydroxy-chol-5-en-24-oyl-CoA (Fig. 2*B*), a cholesterol metabolite with a C_5_ side chain (modified from the natural C_8_ side chain), was approximately two orders of magnitude lower than that for bile acid substrates.

**Table 1:** Apparent substrate specificity profile of *Ct*Sal in the *Ct*Shy–*Ct*Sal complex for hydrated bile acids and cholesterol

Substrate	$K_{m}\left(\mu M\right)$	$k_{cat}\left(s^{-1}\right)$	kcatKm(M−1s−1)
Cholyl-22-OH-CoA	8.12 ± 0.477	37.6 ± 0.808	(4.63 ± 0.289) × 10^6^
Deoxycholyl-22-OH-CoA	8.25 ± 0.660	26.3 ± 0.804	(3.19 ± 0.273) × 10^6^
Chenodeoxycholyl-22-OH-CoA	10.48 ± 0.607	34.5 ± 0.828	(3.29 ± 0.206) × 10^6^
Lithocholyl-22-OH-CoA	4.36 ± 0.476	23.92 ± 1.10	(5.49 ± 0.650) × 10^6^
3β,22-dihydroxy-chol-5-en-24-oyl-CoA	N.D.	N.D.	1.77 × 10^4^^a^

N.D. — not detected due to large *K*_*m*_ values.

a Estimated from the gradient of the v vs. [S] slope given that *K*_*m*_ is too large to measure.

### The crystal structure of *Ct*Shy_**DUF35**_–*Ct*Sal reveals an **αββα** heterotetrameric complex

**Figure 3:**
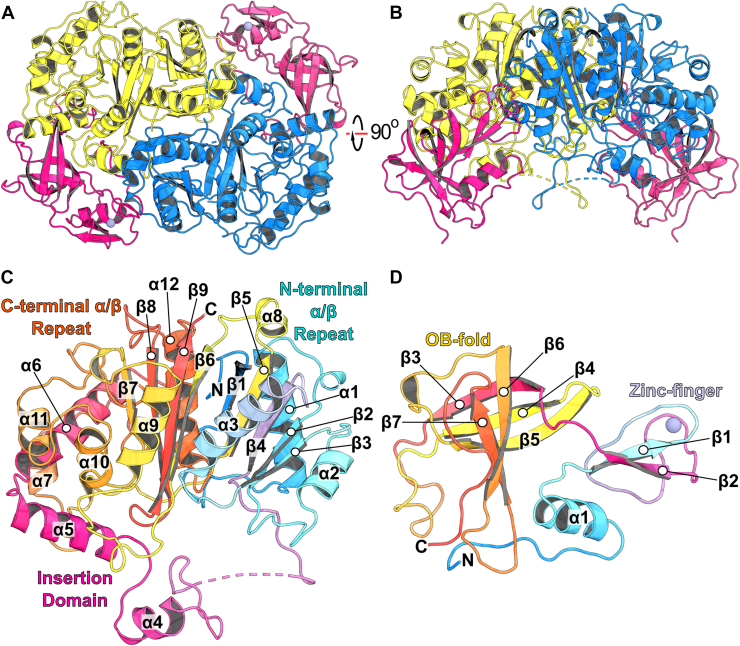
**Crystal Structure of *Ct*Shy_DUF35_–*Ct*Sal.***A* and *B*, orthogonal views of the organization of the heterotetrameric *Ct*Shy_DUF35_–*Ct*Sal complex, with *Ct*Sal colored in *yellow* and *blue*, and the *Ct*Shy_DUF35_ domains colored in *magenta*. The zinc ions in the DUF35 domains are depicted as *gray*–*blue* spheres. *C*, labeled structure of *Ct*Sal colored from *blue* (N-terminus) to *red* (C-terminus). *D*, labeled structure of *Ct*Shy_DUF35_ colored from *blue* (N-terminus) to *red* (C-terminus).

Crystals of the *Ct*Shy_DUF35_–*Ct*Sal complex diffracted to 1.95 Å resolution. Phasing was solved by molecular replacement with a *Ct*Shy_DUF35_–*Ct*Sal heterodimeric model predicted by ColabFold (14) used as the search model. Data collection and refinement statistics are provided in Table 2. The *Ct*Shy_DUF35_–*Ct*Sal complex adopts an αββα heterotetrameric (diprotomeric) architecture, featuring a central *Ct*Sal dimer flanked by two *Ct*Shy_DUF35_ domains, with a total buried interface area of 9540 Å^2^ (*Ct*Sal–*Ct*Sal and *Ct*Shy_DUF35_–*Ct*Sal interfaces; calculated using PISA (15)) (Fig. 3). *Ct*Sal adopts a canonical thiolase fold, consisting of two pseudo-symmetric αβ domains: the N-terminal domain contains a five-stranded mixed β-sheet (topology: 1, −5, 4, 2, 3), while the C-terminal domain harbors a four-stranded mixed β-sheet (topology: 6, −9, 8, 7) (positive integers indicate successive β-strands oriented in one direction, while negative integers indicate β-strands oriented anti-parallel to these) (Fig. 3). Both domains are flanked by α-helices, with α3 and α12 positioned between the β-sheets. In the N-terminal domain, the β4–β5 loop extends into a series of α-helices (α4–α7) and loops that pack against the C-terminal domain. A proximal segment of this loop (residues 127–135) is disordered.

**Table 2:** *Ct*Shy_DUF35_–*Ct*Sal data collection and refinement statistics

Parameter	Statistics
Resolution range	47.09–1.95 (2.02–1.95)
Space group	P2_1_2_1_2_1_
Unit cell	85.46 104.42 112.85 90 90 90
Total reflections	464,657 (45,188)
Unique reflections	72,083 (7033)
Multiplicity	7.8 (6.4)
Completeness (%)	97.2 (95.5)
Mean I/sigma(I)	9.65 (1.13)
Wilson B-factor	23.26
R-meas	0.116 (1.61)
CC1/2	0.997 (0.503)
Reflections used in refinement	66,136 (5366)
Reflections used for R-free	3303 (259)
R-work	0.1812 (0.2852)
R-free	0.2290 (0.3397)
CC(work)	0.957 (0.678)
CC(free)	0.945 (0.675)
Number of non-hydrogen atoms	9186
macromolecules	8153
ligands	2
solvent	1031
Protein residues	1074
RMS(bonds)	0.002
RMS(angles)	0.55
Ramachandran favored (%)	97.0
Ramachandran allowed (%)	3.0
Ramachandran outliers (%)	0.0
Rotamer outliers (%)	1.29
Clashscore	3.59
Average B-factor	27.57
macromolecules	26.79
ligands	22.73
solvent	33.73

Statistics for the highest-resolution shell are shown in parentheses.

The *Ct*Shy_DUF35_ domain comprises an N-terminal zinc finger and a C-terminal oligonucleotide/oligosaccharide-binding (OB) fold (Fig. 3). The zinc finger domain features a double-stranded antiparallel β-sheet (topology: 1, −2) and a single α-helix (α1), while the OB-fold domain consists of a five-stranded β-barrel (topology: 3, −4, 5, −7, 6). The N-terminal helix and OB subdomain of *Ct*Shy_DUF35_ wrap around α4 helix in *Ct*Sal, forming the primary interaction interface between the two proteins.

*Ct*Shy_DUF35_–*Ct*Sal shares closest structural homology with *Tc*ChsH2_DUF35_–*Tc*Ltp2 (PDB 6OK1), with a pairwise RMSD of 1.70 Å between *Ct*Sal and *Tc*Ltp2. Both steroid aldolases adopt a thiolase fold, displaying similar global secondary structure organization. MM-align analysis (16) of the *Ct*Shy_DUF35_–*Ct*Sal and *Tc*ChsH2_DUF35_–*Tc*Ltp2 heterotetramers yielded a global RMSD of 3.31 Å. A DALI search (17) of the Protein Data Bank revealed additional structurally homologous complexes which include the following: benzoylsuccinyl-CoA thiolase from *Geobacter metallireducens* GS-15 (PDB 7PXP; global RMSD of 4.54 Å; pairwise RMSD of 2.7 Å with *Ct*Sal), acetoacetyl-CoA thiolase/HMG-CoA synthase from *Methanothermococcus thermolithotrophicus* (PDB 6ESQ; global RMSD of 4.54 Å; pairwise RMSD of 2.4 Å with *Ct*Sal), and the multi-component Friedel-Crafts acylase PhlABC, which catalyzes C-acylation of phenolic substrates and includes a single DUF35 (PhlB) per thiolase domain (PhlC) from *Pseudomonas protegens* Pf-5 (PDB 5M3K; global RMSD of 3.28 Å with a monomer of *Ct*Shy_DUF35_–*Ct*Sal; pairwise RMSD of 2.4 Å with *Ct*Sal) (Table S1; Fig. S3) (18, 19, 20). In the acetoacetyl-CoA thiolase/HMG-CoA synthase and Friedel-Crafts acylase, the DUF35 domains make further interactions with HMG CoA synthase and PhlA, respectively, providing scaffolds that hold the multienzyme complexes together. The protozoan SCP-2 thiolase-like protein (SLP), which lacks an associated DUF35 domain, also aligns closely with *Ct*Sal (PDB 5AB6; pairwise RMSD of 2.4 Å with *Ct*Sal) (21).

### A novel Cys3His1 zinc finger in *Ct*Shy_**DUF35**_ alters steroid substrate binding in *Ct*Sal

**Figure 4:**
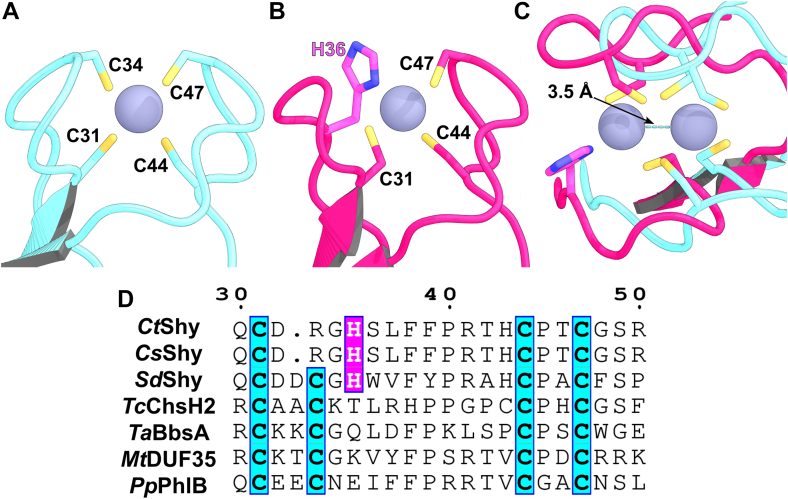
**Comparison of zinc finger in *Ct*Shy_DUF35_, *Tc*ChsH2_DUF35_, and other DUF35 domains.** Residues coordinating zinc ions in the zinc fingers of (*A*) *Tc*ChsH2_DUF35_ (WP_012853804.1; *cyan*) and (*B*) *Ct*Shy_DUF35_ (WP_003057309.1; *magenta*). *C*, structural alignment of *Tc*ChsH2_DUF35_ and *Ct*Shy_DUF35_, showing a separation of 3.5 Å between the zinc ions in each domain. *D*, sequence alignment of *Ct*Shy_DUF35_ and *Tc*ChsH2_DUF35_ with DUF35 domains from homologous thiolase-like complexes, including Shy_DUF35_ from *Comamonas suwonensis* (*Cs*Shy; WP_169108082.1) and *Sterolibacterium denitrificans* (*Sd*Shy; WP_154716410.1), Benzoylsuccinyl-CoA thiolase from *Thauera aromatica* (*Ta*BbsA; AAF89836.1), Acetoacetyl-CoA thiolase/HMG-CoA synthase (*Mt*DUF35; WP_018154495.1), and PhlABC (*Pp*PhlB; ABO30435.1). *Ct*Shy_DUF35_ residue numbering is shown at the *top* of the alignment.

Interestingly, *Ct*Shy_DUF35_ is an outlier among its homologs. It superimposes on its homologs with RMSD values ranging from 1.9 Å (with *Tc*ChsH2_DUF35_) to 2.6 Å. However, while the zinc ion is coordinated by four Cys residues in all homologs including *Tc*ChsH2_DUF35_ (Fig. 4*A*), *Ct*Shy_DUF35_ coordinates the zinc ion with only three Cys ligands (Fig. 4*B*). Specifically, Cys31 maintains the backbone conformation of the equivalent residue in *Tc*ChsH2_DUF35_ but rotates its sidechain by approximately 120° to adopt a new rotamer. In contrast, Cys44 and Cys47, which are part of a loop spanning residues 43 to 51, are positionally shifted when comparing the aligned structures of *Ct*Shy_DUF35_ and *Tc*ChsH2_DUF35_ (Fig. 4*C*). The fourth coordination ligand, His36, completes the coordination sphere by forming interactions with the zinc ion *via* its more sterically accessible Nδ1 atom (Fig. 4*B*). Notably, His36 of *Ct*Shy_DUF35_ does not directly replace Cys34 of *Tc*ChsH2_DUF35_ but instead takes the position of Thr36, with Cys34 being replaced by Arg34 in *Ct*Shy_DUF35_. His36 itself undergoes a ∼1 Å displacement, while the loop encompassing residues 32 to 34 has a single-residue deletion, resulting in further positional adjustments. This substitution transforms the canonical C4-type zinc finger sequence (Cys-x (2)-Cys-x (10)-Cys-x (2)-Cys) into a Cys3His1-type coordination (Cys-x (3)-His-x (8)-Cys-x (2)-Cys) (Fig. 4*D*) (22). This alteration requires the repositioning of the β1–β2 loop, resulting in a 3.5 Å displacement of the zinc ion when the structure of *Ct*Shy_DUF35_ is superimposed with *Tc*ChsH2_DUF35_ (Fig. 4*C*).

The Cys31, Cys44, and Cys47 residues that coordinate zinc in *Ct*Shy_DUF35_ are conserved in other DUF35 homologs. However, the His residue that completes the Cys3His1 coordination is conserved only among DUF35 domains of Shy from various bacteria species but not in DUF35 domains from other enzyme complexes (Fig. 4*D*). In *Ct*Shy_DUF35_, the repositioned zinc ion compacts the β1–β2 loop against the α2 helix in *Ct*Sal, drawing α2 closer to the steroid-binding pocket (Fig. S4). Consequently, the β2–α2 loop protrudes into the active site in *Ct*Sal, occupying the region where the steroid nucleus binds. This structural rearrangement may enhance the binding of bile acids with bent A-ring conformations or C_5_ side chains, thereby optimizing substrate positioning and catalytic efficiency. Such a modification likely contributes to the distinct substrate profile observed in *Ct*Sal, facilitating more effective recognition and processing of steroid substrates.

### *Ct*Sal has flexible loops that shape its extended substrate-binding pocket

The *Ct*Shy_DUF35_–*Ct*Sal crystal structure shows that the active site is an extended pocket formed predominantly from the α/β domains and the insertion domain of *Ct*Sal buttressed by the DUF35 domain, helping form the active site walls on either side (Fig. S5). The *Ct*Shy_DUF35_–*Ct*Sal tetramer has two distinct pockets separated by the β2–α2 loops of both protomers, unlike *Tc*ChsH2_DUF35_–*Tc*Ltp2, where shorter β2–α2 loops create a single long cleft across the *Tc*Ltp2 dimer (Fig. S6).

The 30-residue β4–α4 loop of *Ct*Sal forms part of the wall on either side of the substrate binding site, while its central region (residues 127–135) spans the top of the active site but is disordered. In *Tc*Ltp2, the β4–α4 loop is shorter (18 residues), contributing far less to the walls of the pocket but with a similarly disordered central region (residues 118–127). This contrasts with most thiolase family members, including *Tb*SLP, acetoacetyl CoA thiolase, and PhlC thiolase, which feature an ordered 2- to 3-turn α-helix that forms the active site roof, creating a narrow tunnel for CoA (Fig. S7) (18, 19, 21).

Computational modeling with AlphaFold3 (AF3) (23) predicts that the disordered β4–α4 region in *Ct*Sal likely forms a 10-residue α-helix packing against the β2–α2 loop, leaving the site relatively accessible (Fig. S7*A*). For *Tc*Ltp2, a less structured loop interacting with CoA is predicted, again leaving the distal portion of the active site open (Fig. S7*B*). Interestingly, the equivalent region in benzoylsuccinyl-CoA thiolase is also disordered in the crystal structure (20) and is predicted by AF3 to adopt a conformation resembling that of *Tc*Ltp2 (Fig. S7*C*). These models suggest that *Ct*Sal, *Tc*Ltp2, and benzoylsuccinyl-CoA thiolase have larger, more open substrate-binding pockets than other homologs such as *Tb*SLP (Fig. S7*D*) and that substrate binding may induce a local disorder-to-order transition that enables these enzymes to accommodate bulkier substrates such as steroids.

### *Ct*Sal conserves CoA-binding interactions of homologous *Tb*SLP

*Tb*SLP, the closest known structural homolog of *Ct*Sal with a bound CoA adduct, gives insight into the potential interactions of CoA moieties (PDB: 5AB6) (21). In *Tb*SLP, the CoA accesses the active site through a tunnel formed by insertion loop residues, with its pyrophosphate group stabilized by Arg197 (conserved in *Ct*Sal as Arg216) (Figs. S8, S9). Substitution of Arg216 with alanine (R216A) increased the *K*_*m*_ for cholyl-22-OH-CoA by 5-fold and reduced *k*_*cat*_ by 3-fold, indicating a direct role for this residue in substrate binding (Table 3; Fig. S2*G*). Structural alignment of CoA-bound *Tb*SLP with *Ct*Sal (Fig. S9) suggests additional stabilization of the 3′-phosphate of the CoA moiety in *Ct*Sal from Lys19, located on the opposite side of the binding pocket. The pantetheine moiety is likely stabilized by the backbone carbonyl of Val228 and the side chain amide of Asn162, while the adenine 6-amino group may interact with the backbone carbonyl of Ile227.

**Table 3:** Apparent kinetic parameters for wild-type *Ct*Sal and *Ct*Sal variants with cholyl-22-OH-CoA

*Ct*Sal variant	$K_{m}\left(\mu M\right)$	$k_{cat}\left(s^{-1}\right)$	kcatKm(M−1s−1)
wt	8.12 ± 0.477	37.6 ± 0.808	(4.63 ± 0.289) × 10^6^
Y302F	a	a	a
C304A	N. D.	N. D.	N. D.
C304H	N. D.	N. D.	N. D.
C304S	N. D.	N. D.	N. D.
Y305F	32.1 ± 1.61	4.94 ± 0.0894	(1.54 ± 0.0821) × 10^5^
Y165F	19.1 ± 1.30	8.39 ± 0.212	(4.39 ± 0.319) × 10^5^
R216A	41.4 ± 1.29	11.64 ± 0.150	(2.81 ± 0.00876) × 10^5^

N.D. — not detected; activity was indistinguishable from the baseline absorbance.

a ∗Indicates activity too low to determine Michaelis-Menten parameters. *Ct*SalY302F has a specific activity of 8.26 × 10^−4^ μmol·min^−1^ mg^−1^ with 50 μM of substrate.

### Tyr302 and Cys304 are the catalytic residues in *Ct*Sal

**Figure 5:**
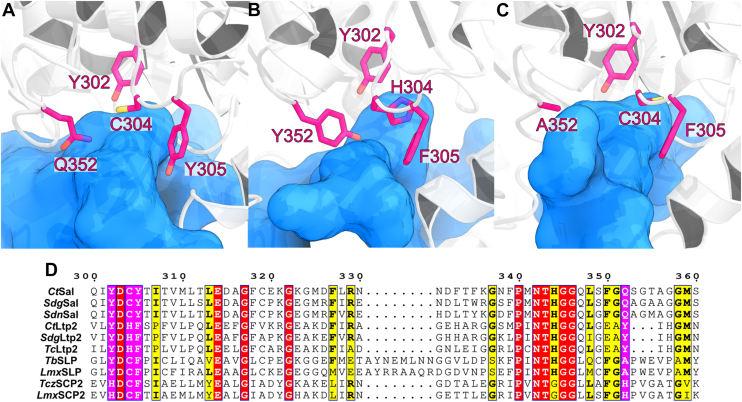
**Comparison of the active sites of *Ct*Sal, *Tc*Ltp2, and *Tb*SLP.** Conserved residues within the active sites of (*A*) *Ct*Sal, (*B*) *Tc*Ltp2, and (*C*) *Tb*SLP are highlighted in *magenta*. These residues are also highlighted in *magenta* in the sequence alignment (*D*), where *Ct*Sal residue numbering is adopted. *Yellow* highlights indicate other partially conserved residues, and *red* highlights indicate other fully conserved residues. The active site cavity is depicted in *blue*. The sources of each sequence in the alignment are provided in Table S4.

While the active site of thiolases is highly conserved, featuring a pair of catalytic cysteine residues, the active sites of homologous thiolase-like enzymes and aldolases are more heterogeneous. The catalytic residues of *Tc*Ltp2 have been identified as Tyr294 (catalytic base in the Y^294^DHF motif) and Tyr344 (catalytic acid; not a part of the YDHF motif). The Y^294^DHF motif of *Tc*Ltp2 is partially conserved as Y^302^DCY in *Ct*Sal and Y^297^DCF in *Tb*SLP (Fig. 5). In contrast, Tyr344 corresponds to Gln352 in *Ct*Sal (Fig. S8).

A Tyr302Phe ((Y→F)^302^DCY) variant abolished *Ct*Sal activity, confirming the importance of Tyr302 in catalysis (Table 3), consistent with findings of the equivalent tyrosine in *Tc*Ltp2. Similarly, Cys304Ala and Cys304Ser variants of the YD(C→A/S)^304^Y motif of *Ct*Sal led to an enzyme with undetectable activity (Table 3). A Cys304His variant (YD(C→H)^304^Y), where His corresponds to the equivalent residue in *Tc*Ltp2 (YDHF), failed to restore activity (Table 3). A Tyr305Phe variant (YDC(Y→F)^305^) in *Ct*Sal resulted in a modest 7.6-fold decrease in *k*_*cat*_ (Table 3). Replacement of Tyr165 (conserved in *Tb*SLP) in the active site with Phe resulted in only a 4.5-fold decrease in *k*_*cat*_ (Table 3). Tyr305 and Tyr165 are therefore unlikely to serve general acid or base roles in the aldolase reaction of *Ct*Sal.

**Figure 6:**
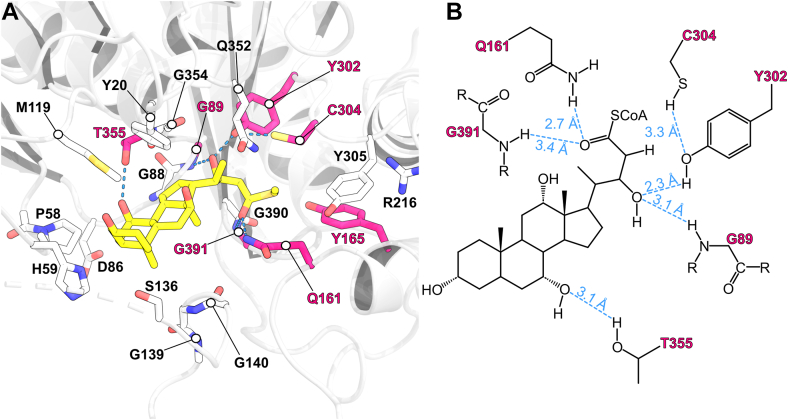
**Cholyl-22-OH-CoA modelled in the *Ct*Sal active site.***A*, cholyl-22-OH-CoA is depicted in *yellow*, while the *Ct*Sal structure is shown in *white*. Residues associated with site-specific mutagenesis kinetic data or steroid binding are represented as magenta sticks and labeled. Predicted hydrogen bonds are indicated by *blue dashed lines*. Residues that lack hydrogen bond contact or those involved in steric packing of the steroid are shown as *white sticks*. *B*, a 2D schematic representation of the modelled substrate, illustrating cholyl-22-OH-CoA in plane with potential hydrogen bonds.

To further investigate the roles of Cys304 and Tyr302 of *Ct*Sal in catalysis, a cholyl-22-OH-CoA substrate was modeled into *Ct*Sal with a minimal thiomethyl moiety standing in for the flexible CoA moiety whose exact conformation is difficult to predict. This molecule was manually positioned within the active site of *Ct*Shy_DUF35_–*Ct*Sal, and minimized in Rosetta 3 (Fig. 6) (24). In this model, the sulfhydryl group of Cys304 is in van der Waals contact with the α-carbon of the steroid side chain, while the phenolic group of Tyr302 forms a hydrogen bond with the Cβ-OH of cholyl-22-OH-CoA (Fig. 6). This suggests that Tyr302 and Cys304 in *Ct*Sal are optimally arranged for acid-base catalysis of the hydroxylated C_5_ steroid side chain.

### AlphaFold modeling of *Ct*Shy–*Ct*Sal reveals a tethered protein complex

**Figure 7:**
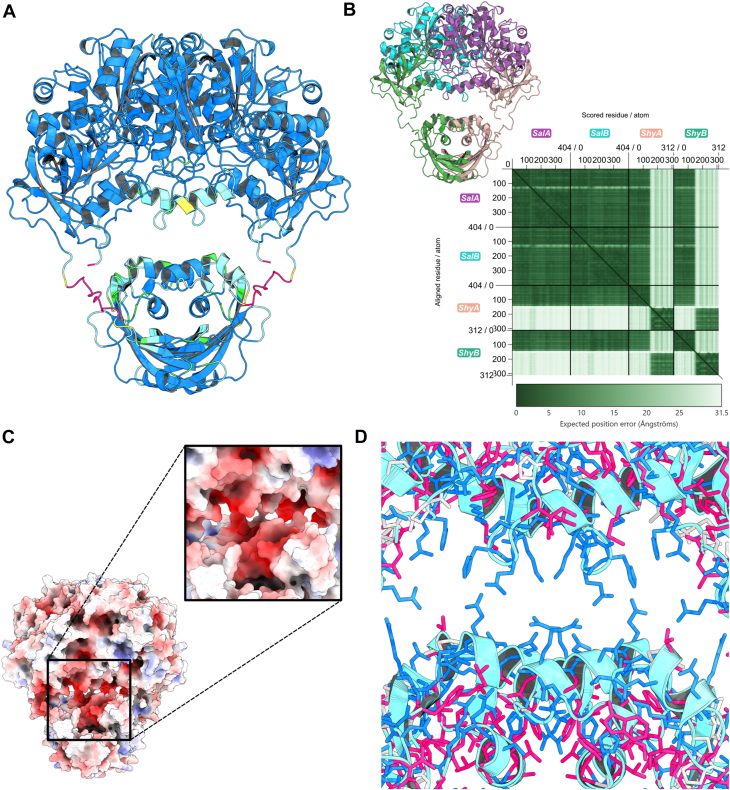
**AlphaFold3 model of the *Ct*Shy–*Ct*Sal complex.***A*, the *Ct*Shy–*Ct*Sal model colored by predicted local distance difference test (pLDDT) scores to indicate confidence in the structural prediction. *B*, the predicted alignment error (PAE, Å) of the model shown by chain, with the inset showing the PAE graph. *C*, the surface view of the interface between the *Ct*Shy and *Ct*Sal dimers is colored by electrostatics, where *red* indicates negatively charged areas, *blue* indicates positively charged areas, and white indicates neutral regions. *D*, the proximal region between *Ct*Shy and *Ct*Sal is colored by hydrophobicity, where *blue* represents hydrophilic residues, *magenta* represents hydrophobic residues, and *white* indicates neutral residues. Secondary structures are shown in *cyan*.

All attempts to crystallize the full *Ct*Shy–*Ct*Sal complex were unsuccessful, so this complex was modeled using AF3 (23) (Fig. 7). The experimentally determined molecular weight of the complex (160 kDa by size exclusion chromatography (13)) aligns closely with a heterotetrameric assembly containing two copies each of *Ct*Shy (35 kDa theoretical) and *Ct*Sal (46 kDa theoretical). *Ct*Shy comprises two domains: *Ct*Shy_DUF35_ and *Ct*Shy_MaoC_ (the latter being the functional hydratase domain). The AF3 model shows strong agreement with experimental structures of *Ct*Shy_DUF35_–*Ct*Sal reported here and *Ct*Shy_MaoC_ that was previously solved (PDB 8VWR (13)) (RMSD = 0.5 Å and 0.7 Å, respectively), with predicted Local Distance Difference Test (pLDDT) scores ≥90 for most regions and low overall predicted alignment error (PAE) (Fig. 7, *A* and *B*). The AF3 model shows a 26-residue linker that was not captured in the experimental structures tethers CtShy_DUF35_ to *Ct*Shy_MaoC_ (13). The structure of this linker is predicted with low confidence (pLDDT <50; Fig. 7*A*), and the PAE between CtShy_MaoC_ and *Ct*Sal is high (Fig. 7*B*). In addition, the interface is loosely packed, and the adjacent faces of both *Ct*Sal and *Ct*Shy_MaoC_ are predominantly electronegative (Fig. 7*C*) and hydrophilic (Fig. 7*D*). This suggests that there is no well-defined interface between these domains, and instead, *Ct*Sal and *Ct*Shy_MaoC_ are tethered by a linker that is intrinsically disordered while the domains are free to move relative to one another. Note that a fully extended tether might allow these domains to separate as much as 40 Å. The depicted complex of *Ct*Shy_MaoC_ and *Ct*Sal should thus be interpreted as a plausible (though likely atypically compact) conformation selected from a broad ensemble, rather than a definitive model.

Despite the absence of a fixed interaction geometry, substrate channeling may conceivably occur if substrate anchored to the CoA-binding site of Shy can reach the active site of Sal, or if the non-polar substrate prefers to move from one domain to the other without fully dissociating during collisions. To test for substrate channeling, we performed a *Ct*Shy–*Ct*Sal coupled assay in the presence of excess 17-β-hydroxysteroid dehydrogenase (Hsd4A) from *Rhodococcus jostii* RHA1 (Fig. S10) (25). Hsd4A catalyzes the NAD^+^-dependent hydroxyl to keto oxidation of hydrated C_5_-steroid side chains produced by *Ct*Shy, sequestering them in a form that is inaccessible to *Ct*Sal. The accumulation of the β-keto form allows for the quantification of the hydrated product released by *Ct*Shy into the bulk solvent, measured spectrophotometrically by monitoring NADH production at 340 nm. The assay showed that 93 ± 1% of the *Ct*Shy product is dehydrogenated by Hsd4A, suggesting that there is no significant substrate channeling between *Ct*Shy and *Ct*Sal.

We further analyzed the products of this assay using LC-MS/MS to verify the reaction products with controls for the *Ct*Shy–*Ct*Sal and Hsd4A reactions (Fig. S11). In the first control, the Hsd4A reaction mixture contained cholyl-enoyl-CoA (the *Ct*Shy substrate) but no *Ct*Shy–*Ct*Sal, and cholyl-enoyl-CoA was the only steroid detected, indicating no substrate turnover (Fig. S11*A*). In the second control, both cholyl-enoyl-CoA and *Ct*Shy–*Ct*Sal were present without Hsd4A, allowing the detection of the *Ct*Sal retroaldol product, 4-pregnen-3-one-20β-carboxaldehyde, along with acetyl-CoA (Fig. S11*B*). When all components, cholyl-enoyl-CoA, *Ct*Shy–*Ct*Sal, and Hsd4A, were present, Hsd4A effectively sequestered the *Ct*Shy product to form cholyl-22-oxo-CoA (Fig. S11*C*), preventing the *Ct*Shy product from reaching *Ct*Sal, and neither 4-pregnen-3-one-20β-carboxaldehyde nor acetyl-CoA was detected.

## Discussion

The proteobacterial steroid aldolase, *Ct*Sal, catalyzes the retroaldol cleavage of the C22–C23 bond of bile acid side chains. The enzyme shows high catalytic efficiency for cholyl-22-OH-CoA, deoxycholyl-22-OH-CoA, chenodeoxycholyl-22-OH-CoA and lithocholyl-22-OH-CoA, which differ only in the number and positions of hydroxyl substituents on the steroid rings (Fig. 2; Table 1). Although Thr355 may form a hydrogen bond with the C7 hydroxyl group (absent in deoxycholate and lithocholate), no significant differences in turnover were observed with substrates missing this functional group. The solvent-exposed positions of the hydroxyl substituents, as inferred from substrate modeling, mitigate any effects on catalytic activity (Fig. 6).

Conversely, *Ct*Sal exhibits about 260-fold reduced catalytic efficiency toward the cholesterol metabolite 3β,22-dihydroxy-chol-5-en-24-oyl-CoA that contains the same C_5_ side chain as the bile acid metabolites (Fig. 2; Table 1). This reduced efficiency suggests that the preference of *Ct*Sal for bile acids over unsaturated cholesterol derivatives is due to differences in the conformation of the steroid ring; cholesterol adopts a planar steroid nucleus that extends linearly along the A-ring, while the A-ring of bile acids is bent out of plane. Our model indicates that a planar steroid ring would clash with His59 from the β2–α2 loop (Fig. 6); interestingly, this loop distinguishes *Ct*Sal and *Tc*Lpt2 by forming a central ridge in *Ct*Sal that divides its long continuous active site cleft into two discrete pockets, whereas this division is not seen in *Tc*Ltp2. The stereoisomer of the α-hydroxyl at C3 of cholesterol differs from the β-epimer found in bile acids at the equivalent position; this may also perturb A-ring binding of cholesterol. The steroid A-ring also interacts with the β4–α4 loop, which is disordered in the experimental structure but is predicted by AF3 to form an α-helix that may occlude cholesterol binding yet help stabilize the sterol rings of the bile acid substrates (Figs. 7, S7). Of note, the order–disorder transition of this sequence creates a structurally dynamic active site volume, an essential evolutionary adaptation that enables *Ct*Sal to bind large acyl-CoA-esterified substrates, including steroids.

Although *Ct*Shy and *Ct*Sal domains have been characterized individually, the rationale for the native assembly of a *Ct*Shy–*Ct*Sal complex remains unclear. In the acetoacetyl-CoA thiolase/HMG-CoA synthase enzyme complex from *M. thermolithotrophicus*, the DUF35 domain mediates tight associations between the thiolase and HMG-CoA synthase (18). The thiolase and HMG-CoA synthase share a CoA binding site at their interface, which facilitates substrate tethering and enables the acetoacetyl chain to transfer efficiently between active sites. Consequently, the acetoacetyl CoA intermediate is not released to the bulk solvent. By comparison, structural modeling of the *Ct*Shy–*Ct*Sal complex suggests that *Ct*Shy and *Ct*Sal are connected *via* relatively long, flexible linkers. The inherent flexibility of this association appears inconsistent with a direct substrate channeling mechanism that transfers cholyl-22-OH-CoA from *Ct*Shy to *Ct*Sal. It will be interesting to determine in the future if substrate channeling occurs in other DUF35 complexes, including ChsH1–ChsH2–Ltp2, benzoylsuccinyl-CoA thiolase and the multi-component Friedel-Crafts acylase PhlABC (11, 19, 20).

**Figure 8:**
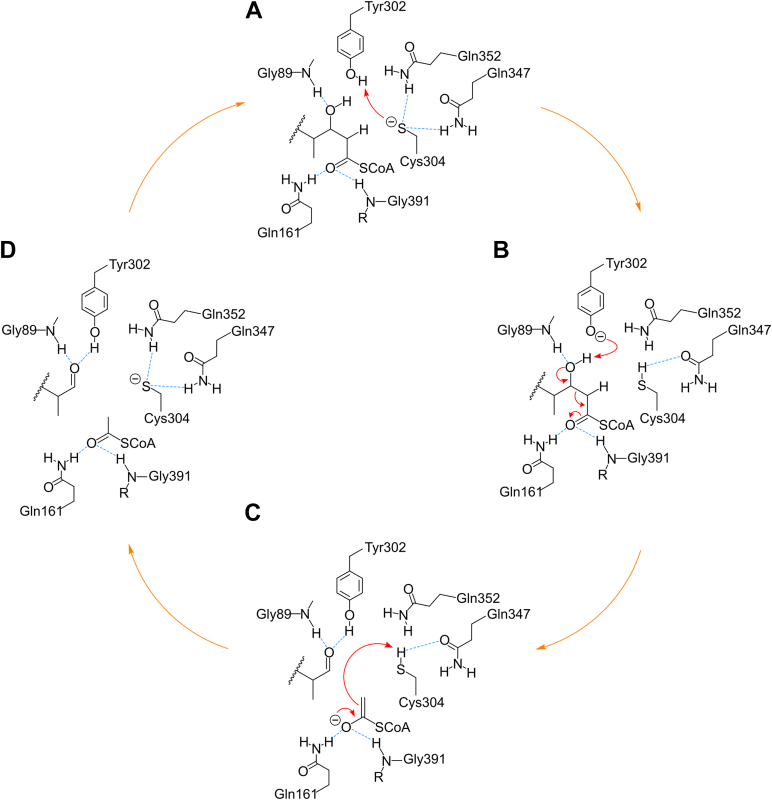
**Proposed catalytic mechanism of *Ct*Sal.** For clarity, the steroid rings are omitted from the figure. *A*, Cys304 may be activated by Gln347 and Gln352, and the thiol anion of Cys304 abstracts a proton from the hydroxyl group of Tyr302. *B*, Tyr302 abstracts a proton from the C17 hydroxyl group of cholyl-22-OH-CoA, initiating cleavage of the C–C bond. *C*, Gln161, and Gly391 form an oxyanion hole, stabilizing the resulting oxyanion intermediate. Cys304 donates a proton to the enolate anion of the CoA thioester. *D*, the release of acetyl-CoA and 4-cholen-3-one-20β-carboxaldehyde.

Cys304 of the YDC^304^Y motif sequence of *Ct*Sal is essential for catalysis, as YD(C→A/S/H)^304^Y mutations abolished activity (Table 3). The undetectable activity with potentially catalytic residues containing catalytic heteroatoms, such as His or Ser, suggests that the thiol group is specifically required for the reaction. Additional mutations of the Tyr residues indicates that only Tyr302 (Y^302^DCY) plays a significant role in catalysis similar to mutational analysis of the equivalent Tyr in the YDHF sequence in *Tc*Ltp2 (12). From substrate modeling, Tyr302 is closest to the C22 hydroxyl group of cholyl-22-OH-CoA (Fig. 6). This hydroxyl group also interacts with the amide backbone of Gly89. An equivalent Gly residue was proposed to form similar interactions to the structurally analogous C17 hydroxyl group in *Tc*Ltp2. Conversely, Tyr165, which we initially hypothesized may serve as a catalytic acid based on its conservation in SCP2-thiolases (Fig. S8) and proximity to the active site (Fig. 6), does not appear to be involved in catalysis in *Ct*Sal, as the Y165F mutant showed only a modest reduction in activity (Table 3; Fig. S2*F*). Taken together, Tyr302 is proposed to act as a catalytic base, abstracting a proton from the C22-hydroxyl of the C_5_ side chain of cholyl-22-OH-CoA, thereby stimulating C–C bond cleavage and producing 4-cholen-3-one-20β-carboxaldehyde with a C_3_ side chain (Fig. 8). Cys304 serves as a catalytic acid to donate a proton to the resulting acyl CoA enolate forming acetyl CoA. Note that both of these residues have relatively high intrinsic *pKa* values. However, the Cys304 thiolate anion may be stabilized by hydrogen bonds with Gln347 and Gln352 (26), and Cys304 and Tyr302 are positioned to form a hydrogen bond network that allows them to cooperate as a catalytic dyad (Fig. 8).

Replacement of the His residue (His 296) in the YDH^296^F motif in *Tc*Ltp2 led to a modest 14-fold reduction in catalytic efficiency, suggesting it was unlikely to be the catalytic acid (12). Substrate docking into the *Tc*Ltp2 structure showed that this His 296 instead participated in forming an oxyanion hole that stabilizes the acyl CoA enolate anion while a tyrosyl residue (Tyr344) is the catalytic general acid (12). Here the oxyanion hole in *Ct*Sal is rather proposed to consist of the backbone amide nitrogen of Gly391 and side chain amide nitrogen of Gln161 (Fig. 6), while Tyr344 of *Tc*Ltp2 is replaced with catalytically inert Gln352 in *Ct*Sal (Fig. S8). Interestingly, the YDCY sequence of *Ct*Sal, with a conserved Cys residue, is more similar to the sequences in SCP-2 thiolases and SLPs (SCP-2 thiolase-like proteins), which have HDCF and YDCF motifs, respectively, than to the YDHF sequence in *Tc*Ltp2 (21, 27).

The function of *Tb*SLP remains unclear. Earlier studies have shown that this enzyme catalyzes the decarboxylation of malonyl-CoA, although with a very low *k*_cat_ value (0.005 s^−1^) (21). To assess potential malonyl decarboxylase activity in *Ct*Sal–*Ct*Shy, *Ct*Sal was incubated with malonyl-CoA and no acetyl-CoA was detected, confirming that *Ct*Sal lacks this catalytic capability. In SCP-2 thiolases, a catalytic Cys residue is proposed to function in a manner analogous to an equivalently positioned residue in *Ct*Sal, that donate a proton to the acyl-CoA enolate intermediate following C–C bond cleavage. Canonical SCP-2 thiolases use the second nucleophilic Cys residue purportedly to attack the 3-ketoacyl-CoA substrate and facilitate thiolytic cleavage. Conversely, *Ct*Sal has a Gly residue positioned equivalently to this catalytic Cys residues in SCP-2 thiolases; thus, *Ct*Sal cannot catalyze thiolytic cleavage of 3-ketoacyl-CoA substrates. More generally, Sals and SLPs do not share any of the five Cys residues found in SCP-2 thiolases (21).

The YDCY motif in *Ct*Sal aligns closely with YDCF in *Tb*SLP both in sequence and in structural position. These enzymes globally share a similar active-site architecture, including comparable α-helices within the β4–α4 loop. Given that the catalytic residues identified in *Ct*Sal are conserved in *Tb*SLP, the structural similarities between *Ct*Sal and *Tb*SLP, the unresolved substrate specificity of *Tb*SLP, and its low activity with canonical thiolase substrates, we propose that *Tb*SLP may be an aldolase rather than a thiolase-like protein. Future studies could explore this hypothesis by screening substrates containing a β-hydroxy carbonyl group to evaluate their activity with *Tb*SLP, potentially leading to the reclassification of SLPs as aldolases, with important implications for understanding their role in lipid metabolism and their evolutionary history.

**Figure 9:**
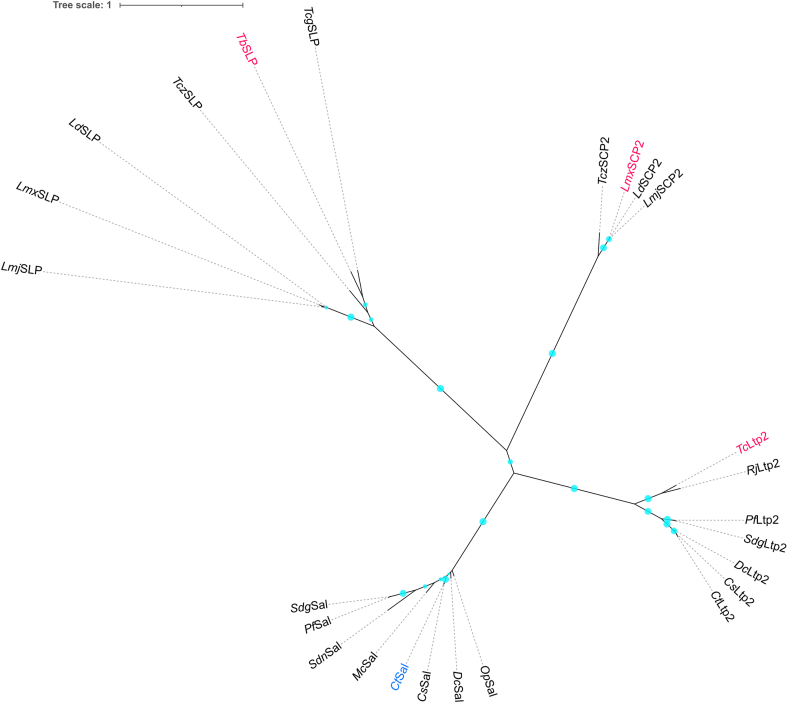
**An unrooted maximum likelihood dendrogram depicting the relationships among Sals, Ltp2s, SLPs, and SCP2 enzymes.***Cyan dots* on the branches indicate bootstrap values of ≥ 50%. Previously characterized representative proteins are shown in *magenta*, while *Ct*Sal from this study is highlighted in *blue*. The sources of protein sequences, along with the full enzyme and species names, are provided in Table S4.

Phylogenetic assessment reveals that Sals, Ltp2s, SLPs, and SCP-2 thiolases form distinct clades (Fig. 9). It is possible that the catalytic Cys residue in the XDCX pattern is found in the common ancestor of these enzymes. Overall, the similarity of active sites in *Ct*Sal and *Tb*SLP suggests that the core elements might represent an ancestral state. Interestingly, several distinct families of steroid degradation enzymes (including hydratases and enoyl reductases) appear to accommodate different chain-length substrates by utilizing conserved catalytic machinery and positioning the steroid ring in the active site to align the reactive centre with the appropriate catalytic residues (13, 28). In contrast, Ltp2s seem to have repurposed the Sal-family active site for C_3_ side chains not by repositioning the substrate, but rather by employing a largely new set of catalytic residues that extend closer to the C17-OH group of hydrated C_3_ side chains. Possibly, with the hydroxyl group located on C17 of the steroid ring, and a methyl substituent on C20, C_3_ side chain substrates are too sterically encumbered to bind productively in a Sal-like catalytic site.

While Proteobacterial *Ct*Sal homologs are involved in bile acid side chain degradation, *Ct*Sal homologs with a YDCY sequence also exist in cholesterol degrading Actinobacteria (10, 29). Future structure-function studies of these enzymes will clarify how their active sites accommodate steroids with distinct ring architectures. These bacterial steroid aldolases may serve as biocatalytic tools for pharmaceutical production, enabling selective cleavage of side chains to generate novel derivatives with enhanced therapeutic properties while streamlining synthesis and reducing reliance on harsh chemical reagents.

## Experimental procedure

### Chemicals

Restriction enzymes and Pfu polymerase were sourced from Thermo Scientific, while T4 DNA ligase was obtained from New England Biolabs. Ni^2+^-NTA Superflow resin was acquired from Qiagen. Cholic acid was obtained from Alfa Aesar. Chenodeoxycholic acid, deoxycholic acid, and lithocholic acid were purchased from Sigma-Aldrich. 3β-hydroxy-Δ^5-^cholenic acid was acquired from Tokyo Chemical Industry Co., Ltd. Coenzyme A was procured from BioShop Canada Inc., and ATP was acquired from Bio Basic Inc. All other chemicals were obtained from Thermo Scientific or Sigma-Aldrich unless specified otherwise.

### Bacterial strains and plasmids

*C. testosteroni* KF-1 was acquired from DSMZ-German Collection of Microorganisms and Cell Cultures, while *E. coli* BL21 LOBSTR was obtained from Kerafast Inc.

### DNA manipulation

DNA purification, digestion, and ligation were performed using standard protocols. The genes for *ctshy* and *ctsal* were previously inserted into the *E. coli* expression plasmids pBLTlactac and pMCSG7 (13). Site-directed mutagenesis was carried out using a modified QuikChange method with primers listed in Table S2 (30). All plasmids were cloned in *E. coli* DH5α (Invitrogen) following transformation. Validation of cloned genes and mutations was confirmed through DNA sequencing at Laboratory Services, University of Guelph. The plasmids were then introduced into *E. coli* BL21 LOBSTR for expression (31).

### Protein expression, purification, and analysis

Recombinant *E. coli* cultures were grown in 4 L of LB media supplemented with ampicillin (100 μg/ml) and tetracycline (15 μg/ml) at 37 °C. When the cultures reached mid-log phase (OD_600_ of 0.4–0.6), recombinant protein expression was induced by adding 1 mM isopropyl-β-D-thiogalactopyranoside (IPTG). The cells were then incubated for an additional 24 h at 15 °C and harvested by centrifugation at 9605*g* for 10 min. The *E. coli* cell pellets were resuspended in 20 mM HEPES buffer (pH 7.5) and lysed by passing through a French press at 103,421 kPa. Afterward, the cell lysates were centrifuged at 39,191*g* for 20 min. The resulting cell extracts were filtered through a 0.45-μm filter and then incubated for 1 h at 4 °C with Ni^2+^-NTA resin in buffer (20 mM HEPES, pH 7.5) containing 20 mM imidazole (pH 8.0). Subsequently, the mixture was poured into a gravity column and washed with the same buffer. The His-tagged proteins were eluted using buffer containing 150 mM imidazole (pH 8.0). The eluted proteins were then concentrated to a final volume of 0.5 to 1 ml and exchanged back to 20 mM HEPES buffer (pH 7.5) by dilution in a stirred cell equipped with a YM10 filter (Amicon). The purified enzymes were stored at −80 °C. Protein concentrations of the purified samples were determined by Bradford assays, using bovine serum albumin as a standard (32). Purity of the enzyme purifications was evaluated by Coomassie blue-stained SDS-PAGE.

### Differential scanning fluorimetry

The melting temperature of both wild-type *Ct*Shy–*Ct*Sal and its variants was assessed using SYPRO Orange, and fluorescence was monitored using the StepOnePlus Real-Time PCR system (Applied Biosystems) (Table S3). These experiments were conducted in triplicate, with a protein concentration of 0.3 mg/ml in 20 mM HEPES buffer at pH 7.5 and 1X Sypro Orange, following established protocols (33).

### Cholyl-22-OH-CoA, deoxycholyl-22-OH-CoA, and chenodeoxycholyl-22-OH-CoA synthesis

Cholyl-22-OH-CoA, chenocholyl-22-OH-CoA, and chenodeoxycholyl-22-OH-CoA were synthesized following established procedures (13, 28). Briefly, in a 10 ml reaction mixture containing 100 mM sodium HEPES buffer (pH 7.5), bile acid (1.0 mM), CoA (1.0 mM), ATP (2.5 mM), magnesium sulfate (5.0 mM), and the acyl-CoA synthetase CasG from *R. jostii* RHA1 (34) (1.5 μM) were incubated overnight at room temperature (22 °C) with gentle agitation. The produced CoA esterified bile acid was then dehydrogenated and hydrated using the acyl-CoA dehydrogenase CasC from *R. jostii* RHA1 (35) (0.5 mM), potassium hexacyanoferrate (III) (300 μM), and ChsH3 from *Mycobacterium tuberculosis* (28) (1 μM) in 40 ml of 100 mM sodium HEPES buffer (pH 7.5) for 2.5 h at room temperature with gentle agitation. The reaction was stopped by acidification to pH 4.0 with HCl, and the solution was passed through 0.45 μm and YM10 filters. Approximately half of the filtrate (∼20 ml) was loaded onto a 2.8 ml HyperSep Disposable C18 column (500 mg bed weight; Thermo Scientific) equilibrated with 10% acetonitrile in 50 mM sodium phosphate buffer (pH 5.3). The column was washed with the same buffer, and CoA-esters were eluted with 10 ml of 40% acetonitrile in 50 mM sodium phosphate buffer (pH 5.3). This purification process was repeated twice to purify all the filtrate. Acetonitrile was evaporated overnight under a stream of air.

### 3**β**,22-dihydroxy-chol-5-en-24-oyl-CoA and lithocholyl-22-OH-CoA synthesis

3β-hydroxy-Δ5-cholenoic acid and lithocholate were CoA-esterified *via* a mixed anhydride reaction due to the limited water solubility of the parent compounds, using protocols described previously (36). A total of 0.13 mmol of each steroid and 0.26 mmol of triethylamine were dissolved in 6 mL of methylene chloride. A solution of 0.26 mmol of ethyl chloroformate in 2 ml of dichloromethane was added to the dissolved steroid acid, and the mixture was shaken occasionally at room temperature for 1.5 h, covered with parafilm, followed by an additional 30 min with air exposure. The dichloromethane was evaporated, and the resulting anhydride was dissolved in 5 mL of THF. CoA (0.06 mmol) in 5 ml of 0.5 M sodium bicarbonate was added to the mixed anhydride. The solution was then acidified to pH 5.0 with 10% perchloric acid, and THF was removed by evaporation under air for 1 h. Thioesters were precipitated by the addition of 1 ml of 10% perchloric acid and collected by centrifugation, followed by washing with 1 ml of ethyl ether. The produced CoA esters were then dehydrogenated and hydrated using the acyl-CoA dehydrogenase CasC from *R. jostii* RHA1 (35) (0.5 nM) and potassium hexacyanoferrate (III) (300 μM) in 40 ml of 100 mM sodium HEPES buffer (pH 8.5) and 1.0 μM ChsH3 from *M. tuberculosis* (28) for 2.5 h at room temperature with gentle agitation. The resulting hydrated product was purified using the same procedure employed for cholyl-22-OH-CoA described above, with equilibration/wash and elution buffers containing 20% and 80% acetonitrile, respectively.

### Steady-state kinetic assays

Aldolase activity was measured in 1 ml reactions containing 1 mM NAD^+^, 7 nM *Comamonas testosterni* KF-1 *Ct*Sad (*C. testosteroni* steroid aldehyde dehydrogenase) and varying concentrations of steroid substrates in 100 mM sodium HEPES buffer (pH 7.5) at 25 °C. The activity was determined by monitoring the increase in absorbance at 340 nm using a Varian Cary 3 spectrophotometer, corresponding to the reduction of NAD^+^ to NADH (with an extinction coefficient ε340 of 6.22 mM^−1^cm^−1^) due to the transformation of the aldehyde product of *Ct*Sal to a carboxylate by *Ct*Sad. Data was fitted to the Michaelis-Menten equation using nonlinear regression with GraphPad Prism software.

### Protein crystallization and X-ray crystallography data acquisition

Crystallization conditions of *Ct*Shy_DUF35_–*Ct*Sal were screened utilizing the JCSG-plus kit from Molecular Dimensions. Crystals suitable for subsequent data collection were successfully grown in 0.2 M potassium citrate tribasic monohydrate and 20% w/v PEG 3350 *via* the sitting drop method at room temperature (22 °C), with *Ct*Shy_DUF35_–*Ct*Sal concentration maintained at 12 mg/ml. The crystals were cryoprotected by coating crystals with Paratone-N before freezing. Data were collected at the Canadian Macromolecular Crystallography Facility (CMCF-BM) within the Canadian Light Source. Subsequent processing and scaling of datasets were carried out using XDS and XSCALE, respectively (37). Molecular replacement in Phaser utilized a Colabfold model of the *Ct*Shy_DUF35_–*Ct*Sal heterodimer as a search model (14). Refinement was conducted employing PHENIX refine, with manual model building performed using the Crystallographic Object-Oriented Toolkit (Coot) (38, 39). Visualizations of protein structures were generated using PyMOL version 2.0.

### *Ct*Shy–*Ct*Sal substate channeling competition assay

End point assays were conducted in triplicate by incubating *Ct*Shy–*Ct*Sal complex (3.63 nmol), excess Hsd4A (20.8 nmol), cholyl-enoyl CoA (0.50 mM), and NAD^+^(1.00 mM) in 100 mM sodium HEPES buffer (pH 7.5) at 25 °C. The total amount of NADH produced, indicative of the dehydrogenation of the *Ct*Shy product, cholyl-22-OH CoA, released in bulk solvent was measured spectrophotometrically at 340 nm in an endpoint assay.

### LC-MS/MS analysis of substrate channeling products

Reactions were performed as described in the competition assay, except using 50 mM ammonium acetate (pH 7.4) in place of HEPES to improve ionization efficiency. Reaction products were analyzed using a Waters Synapt G2-Si HDMS mass spectrometer coupled to an Acquity UPLC system at the Mass Spectrometry Facility of the Advanced Analysis Centre, University of Guelph. Chromatographic separation was performed on a C18 column (*e.g.*, Waters BEH C18, 50 mm × 2.1 mm, 1.7 μm) with a 10 μl sample volume, using solvent A (water with 0.1% (v/v) formic acid) and solvent B (acetonitrile with 0.1% (v/v) formic acid). The gradient started at 5% B, ramped to 100% B over 15 min, followed by a column wash at 100% B and a 10 min re-equilibration. The flow rate was 0.4 ml/min. The mass spectrometer was operated in negative-ion electrospray ionization mode with the following settings: capillary voltage of 4.0 kV, cone voltage of 40 V, source temperature of 120 °C, desolvation gas temperature of 250 °C, desolvation gas flow of 800 L/h, and nebulizer pressure of 30 psi. Data were acquired in data-dependent MS/MS mode, selecting two precursor ions per cycle for fragmentation across an m/z range of 25 to 2000. Data were processed using Waters Connect mass spectrometry software.

### Protein cavity detection

Cavity analysis was performed using CavitOmiX (version 1.0, 2022, Innophore GmbH). The hydrophobicity within these cavities was evaluated using the hydrophobicity module of the VASCo software (40). Cavity dimensions were determined employing a modified LIGSITE algorithm (41).

### Phylogenetic analysis

Sequences of *Ct*Sal homologs were retrieved from the NCBI database (42) using BLASTp with default parameters (sequence sources listed in Table S4). Multiple sequence alignment of the homologs was performed using Clustal Omega (43) with default settings to ensure accurate alignment of conserved regions. A phylogenetic tree was constructed using the maximum likelihood method implemented in IQ-TREE (44), with the substitution model automatically selected based on the Bayesian Information Criterion (BIC). Bootstrap analysis was performed with 1000 replicates to assess the robustness of the tree topology. The resulting unrooted dendrogram was visualized and annotated using the Interactive Tree of Life (iTOL) tool (45).

## Data availability

The structure of the *Ct*Shy_DUF35_–*Ct*Sal complex has been deposited in the Protein Data Bank under accession code 9MYR. Raw crystallographic data have been deposited in the Zenodo repository under the title “Sal-Shy(DUF35)_9MYR raw crystallographic dataset” (DOI: 10.5281/zenodo.14775288).

## Supporting information

This article contains supporting information.

## Conflict of interest

The authors declare that they have no conflicts of interest with the contents of this article.

MS Kimber is an editorial board member of the *Journal of Biological Chemistry* and was not involved in the editorial review of this manuscript.
