# Supplementary material for: Sal is a proteobacterial bile acid aldolase that repurposes key thiolase catalytic residues for retroaldol cleavage of C5 steroid side chains
Source: J Biol Chem. 2025 Jul 1;301(8):110439. doi: 10.1016/j.jbc.2025.110439 (PMC12329534; doi:10.1016/j.jbc.2025.110439)
Supplement: Supporting information [file mmc1.docx]

**Figure S1. Structure of the steroid cholate.** The bile acid cholate with carbon numbering (C1–C24) and labeled steroid rings (A–D).

**Figure S2. Michaelis-Menten kinetic curves for *Ct*Sal assays.** Kinetic curves for *Ct*Sal in the *Ct*Shy–*Ct*Sal complex are shown for assays with (*A*) Cholyl-22-OH-CoA, (*B*), deoxycholyl-22-OH-CoA, (*C*) chenodeoxycholyl-22-OH-CoA and (*D*) lithocholyl-22-OH-CoA. Curves for assays of *Ct*Sal variants with cholyl-22-OH-CoA are shown for (*E*) *Ct*SalY305F, (*F*) *Ct*SalY165F and (*G*) *Ct*SalR216A. Each reaction was performed in triplicate.

**Figure S3.** **DUF35 Complexes that share homologous domains with *Ct*Shy_DUF35_**–***Ct*Sal.** Each panel shows DUF35 domains in pink. *A*, *Ct*Shy_DUF35_–*Ct*Sal. *B*, *Tc*Ltp2–*Tc*ChsH2_DUF35_ (PDB: 6OK1; RMSD: 2.9 Å). *C*, Benzoylsuccinyl-CoA thiolase (PDB: 7PXP; RMSD: 2.7 Å). *D*, Acetoacetyl-CoA thiolase/HMG-CoA synthase (PDB: 6ESQ; RMSD: 2.4 Å). *E*, Multi-component Friedel-Crafts acylase PhlABC (PDB: 5M3K; RMSD: 2.4 Å).

**Figure S4. Repositioning of the zinc finger in *Ct*Shy_DUF35_ shifts α2 and the β2–α2 loop in *Ct*Sal closer to the catalytic site compared to homologous *Tc*Ltp2.** *Ct*Shy_DUF35_ from the *Ct*Shy_DUF35_–*Ct*Sal complex is shown in magenta, and ChsH2_DUF35_ from the *Tc*ChsH2_DUF35_–*Tc*Ltp2 complex is shown in cyan. The Cys3His1 zinc finger in *Ct*Shy_DUF35_ repositions the zinc ion and the surrounding β1–α1 loop, drawing them closer to the aldolase. As a result, the α2 helix in *Ct*Sal (yellow) is shifted ~7 Å toward the catalytic site relative to α2 in *Tc*Ltp2 (orange). This movement also causes the β2–α2 loop in *Ct*Sal to protrude deeper into the catalytic site than in TcLtp2, positioning it closer to the pocket where the steroid rings, particularly the distal A-ring, would bind. The bent A-ring of bile acid substrates in *Ct*Sal is less likely to clash with the repositioned β2–α2 loop, in contrast to the flatter, more extended cholesterol substrates preferred by *Tc*Ltp2.

**Figure S5. Organization of the *Ct*Shy_DUF35_–*Ct*Sal active site cleft.** The *Ct*Sal active site pocket (blue) lies at the base of a deep cleft formed primarily by its N-terminal (red) and C-terminal (yellow) α/β repeat domains, which together create a concave surface lining the pocket’s floor and walls. The insertion domain (cyan) contributes additional structural elements near the entrance, while the DUF35 domain (magenta) from *Ct*Shy is positioned behind the insertion domain, helping to shape the overall architecture of the active site.

**Figure S6. Comparison of inter-subunit cavities in *Ct*Shy_DUF35_–*Ct*Sal and *Tc*ChsH2_DUF35_–*Tc*Ltp2.** (*A*) In the *Ct*Shy_DUF35_–*Ct*Sal complex (surface view), the β2–α2 loops (shown in red) of both *Ct*Sal protomers extend toward the dimer interface, effectively pinching off the active site pockets of each subunit and resulting in two distinct catalytic pockets. (*B*) In contrast, the *Tc*ChsH2_DUF35_–*Tc*Ltp2 complex features shorter and more compact β2–α2 loops, opening up the active site and allowing the individual active site pockets of each *Tc*Ltp2 protomer to merge into a single, continuous inter-subunit cavity.

**Figure S7. Alphafold3-predicted model of the β4**–**α4 loop in *Ct*Sal and comparison with homologous structures.** The disordered β4–α4 loop, shown in color, which lacked electron density in the experimental structures of (*A*) *Ct*Sal (yellow), (*B*) *Tc*Ltp2 (orange), and (*C*) Benzoylsuccinyl-CoA thiolase (green), was modeled using Alphafold3. For comparison, experimental structures with resolved β4–α4 densities are shown for (*D*) *Tb*SLP (PDB: 5AB4; pink), (*E*) Acetoacetyl-CoA thiolase (PDB: 6ET9; blue), and (*F*) PhlC (PDB: 5M3K; cyan). Catalytic acid/base residues (shown as sticks) and the active site cavities are colored to match the β4–α4 loop in each enzyme.

**Figure S8. Multiple sequence alignment of Sal homologs (shown on previous page).** The aligned amino acid sequences are listed as follows: *Comamonas testosteroni* Sal (*Ct*Sal); *Stutzerimonas degradans* (*Sdg*Sal); *Steroidobacter denitrificans* Sal (*Sdn*Sal); *Comamonas testosteroni* Ltp2 (*Ct*Ltp2); *Stutzerimonas degradans* Ltp2 (*Sdg*Ltp2); *Thermonospora curvata* Ltp2 (*Tc*Ltp2); *Trypanosoma brucei* SLP (*Tb*SLP); *Leishmania Mexicana* SLP (*Lmx*SLP); *Trypanosoma cruzi* SCP2 (*Tcz*SCP2); *Leishmania Mexicana* SCP2 (*Lmx*SCP2). Residue numbering in the alignment corresponds to the *Ct*Sal sequence. For comparative purposes, residue numbers in homologous proteins have been adjusted to align with *Ct*Sal. The following residues are highlighted: Y165 (*green*), R216 (*orange*), Y302 (*purple*), C304 (*blue*), Y305 (*brown*) and Q352 (*cyan*). Yellow highlights indicate other partially conserved residues and red highlights indicate other fully conserved residues.

**Figure S9. Potential Residues Involved in CoA Binding in *Ct*Sal.** Structural alignment of acetylacetyl-CoA-bound *Tb*SLP (PDB: 5AB6) with *Ct*Sal was performed to identify residues in *Ct*Sal positioned within hydrogen-bonding distance of CoA. The analysis suggests that the backbone of Ile227 stabilizes the 6-amino group of the adenine base. The pyrophosphate groups are stabilized through interactions with Lys19 and Arg216, while the backbone carbonyl group of Val228 and the side-chain amide of Asn162 contribute to the stabilization of the pantetheine moiety.

**Figure S10. Reaction schematic for the *Ct*Shy**–***Ct*Sal substrate channelling assay.** To assess substrate channelling in *Ct*Shy–*Ct*Sal, the enzyme complex was incubated with Hsd4A, along with a known concentration of cholyl-enoyl CoA and NAD^+^. The endpoint concentration of cholyl-22-oxo-CoA was quantified by measuring the increase in NADH absorbance at 340 nm. The hydration of cholyl-enoyl CoA by *Ct*Shy is essential for the subsequent C–C bond cleavage catalyzed by *Ct*Sal. Conversely, the Hsd4A product, cholyl-22-oxo-CoA, cannot undergo this reaction. The observed absorbance increase indicates the proportion of *Ct*Shy-generated product released into the bulk solvent and subsequently turned over by Hsd4A.

**Figure S11 (see image on previous page). LC-MS/MS analysis of *Ct*Shy-*Ct*Sal substrate channeling assay products.** Assays were conducted in 50 mM ammonium acetate buffer (pH 7.4) containing cholyl-enoyl-CoA (0.50 mM) and NAD⁺ (1.00 mM). In the presence of Hsd4A (20.8 nmol), the LC trace showed a peak at (*A*) 7.97 min with an m/z of 1154, corresponding to cholyl-enoyl-CoA. When *Ct*Shy–*Ct*Sal (3.63 nmol) was used instead, LC peaks were observed at (*B*) 2.05 and 8.77 min with m/z values of 808 and 363, respectively, corresponding to 4-pregnen-3-one-20β-carboxaldehyde and acetyl-CoA. Cholyl-22-oxo-CoA was not detected in this sample. In the assay containing both Hsd4A (20.8 nmol) and *Ct*Shy–*Ct*Sal (3.63 nmol), a peak at (*C*) 7.55 min with an m/z of 1172 was observed, consistent with the formation of cholyl-22-oxo-CoA. Neither 4-pregnen-3-one-20β-carboxaldehyde nor acetyl-CoA were detected in this sample.

**Table S1: Structural alignment statistics for the top hits from the DALI PDB search of *Ct*Shy_DUF35_–*Ct*Sal homologs.**

| Alignment | Z-score | RMSD (Å) | % sequence identity* |
| --- | --- | --- | --- |
| benzoylsuccinyl-CoA thiolase | 44.6 | 2.7 | 25 |
| acetoacetyl-CoA thiolase/HMG-CoA synthase | 49.3 | 2.4 | 23 |
| PhlABC | 46.2 | 2.4 | 22 |
| TbSLP | 49.1 | 2.4 | 26 |

*% sequence identity calculated over the structurally aligned region between the query (chain A of *Ct*Sal) and the matched structure.

**Table S2: Primers used for mutagenesis of *Ct*Sal.**

| Sal primer | Primer sequence |
| --- | --- |
| C304A | GATCTACGAC**GCC**TACACGATCACCGTGATGCTGACGCTGG  GATCGTGTA**GGC**GTCGTAGATCTGCGCCATATGCATGTCGGAAGG |
| C304H | GATCTACGAC**CAC**TACACGATCACCGTGATGCTGACGCTGG  GATCGTGTA**GTG**GTCGTAGATCTGCGCCATATGCATGTCGGAAGG |
| C304S | GATCTACGAC**TCC**TACACGATCACCGTGATGCTGACGCTGG  GATCGTGTA**GGA**GTCGTAGATCTGCGCCATATGCATGTCGGAAGG |
| Y302F | CGCAGATC**TTC**GACTGCTAC ACGATCACCGTGATGCTGACGC  GTAGCAGTC**GAA**GATCTGCGCCATATGCATGTCGGAAGGGCGC |
| Y305F | TACGACTGC**TTC**ACGATCACCGTGATGCTGACGCTGGAAG  GTGATCGT**GAA**GCAGTCGTAGATCTGCGCCATATGCATGTCGG |
| Y165F | CGCGACTCGTTAGTACCGC**TTC**GGCCACAAGACGC  CATGATTGCTCAGCGCTACGGCGCGGTCCATGG |
| R216A | GCTGAACTCG**GCC**ATGGTGGCCGACCCTCTG  GGCCGACCCTCTGCATGTACTGGAAATCGTGCTGCCG |

*The nucleotide sequences for the forward and reverse *Ct*Sal primers with mutant codons shown in red.

**Table S3: Thermal shift assay results for wt *Ct*Sal and *Ct*Sal variants.**

| Sal variant | Tm (°C) | ΔTm (°C) |
| --- | --- | --- |
| wt | 44.2 ± 0.519 | - |
| C304A | 44.2 ± 0.519 | 0 |
| C304H | 44.5 ± 0.519 | 0.3 |
| C304S | 44.5 ± 0.519 | 0.3 |
| Y302F | 44.5 ± 0.519 | 0.3 |
| Y305F | 43.1 ± 0.519 | 1.1 |
| Y165F | 44.2 ± 0.519 | 0 |
| R216A | 44.1 ± 0.471 | 0.1 |

**Table S4: List of aldolase, thiolase, and thiolase-like sequences used in the dendrogram and multiple sequence alignment.**

| Aldolase/  thiolase | Species | Abbreviated name | Refseq accession number/GeneBank | |
| --- | --- | --- | --- | --- |
| Sal |  |  | |  |
|  | *^a^Comamonas testosteroni* | *Ct*Sal | | WP_003057311.1 |
|  | *Comamonas suwonensis* | *Cs*Sal | | WP_169108081.1 |
|  | *^a^Stutzerimonas degradans* | SdgSal | | ADM35101.1 |
|  | *Pseudomonas flexibilis* | *Pf*Sal | | WP_039606914.1 |
|  | *^a^Sterolibacterium denitrificans* | *Sdn*Sal | | WP_154716409.1 |
|  | *Oryzisolibacter propanilivorax* | *Op*Sal | | WP_091565263.1 |
|  | *Massilia cavernae* | *Mc*Sal | | WP_119810486.1 |
|  | *Diaphorobacter caeni* | *Dc*Sal | | WP_194773065.1 |
| Ltp2 | *^a^Comamonas testosteroni* | *Ct*Ltp2 | | WP_302093115.1 |
|  | *Comamonas suwonensis* | *Cs*Ltp2 | | WP_169108140.1 |
|  | *Rhodococcus jostii* | *Rj*Ltp2 | | WP_073364101.1 |
|  | *^a^Stutzerimonas degradans* | *Sdg*Ltp2 | | EKM95662.1 |
|  | *Pseudomonas flexibilis* | *Pf*Ltp2 | | WP_039606934.1 |
|  | *^a^Thermomonospora curvata* | *Tc*Ltp2 | | WP_012853801.1 |
|  | *Diaphorobacter caeni* | *Dc*Ltp2 | | MBF5004300.1 |
| SLP | *^a^Trypanosoma brucei* | *Tb*SLP | | XP_011775472.1 |
|  | *Trypanosoma congolense* | *Tcg*SLP | | CCC91956.1 |
|  | *Trypanosoma cruzi* | *Tcz*SLP | | KAF8294621.1 |
|  | *^a^Leishmania mexicana* | *Lmx*SLP | | XP_003873552.1 |
|  | *Leishmania major* | *Lmj*SLP | | XP_001681942.1 |
|  | *Leishmania donovani* | *Ld*SLP | | XP_003859553.1 |
| SCP2 | *^a^Trypanosoma cruzi* | *Tcz*SCP2 | | XP_807246.1 |
|  | *^a^Leishmania mexicana* | *Lmx*SCP2 | | XP_003875706.1 |
|  | *Leishmania major* | *Lmj*SCP2 | | XP_001683404.1 |
|  | *Leishmania donovani* | *Ld*SCP2 | | XP_003860986.1 |

*^a^*indicates sequences used in the multiple sequence alignment in Fig. S1.
